# Two Cases of Severe Preeclampsia Were Diagnosed with HELLP Postpartum after Caesarian Section

**DOI:** 10.1155/2014/747510

**Published:** 2014-07-20

**Authors:** Xuechuan Han, Yang Fan, Yan Yu

**Affiliations:** Department of Gynecology and Obstetrics, Ningxia People's Hospital, Yinchuan, China

## Abstract

HELLP occurs in 0.5%–0.9% of all pregnancies. About 30% of the cases happen within 48 hours after delivery. Women with postpartum HELLP syndrome have significantly higher incidences of complications. Because of the absence of classical signs of preeclampsia, it can confuse physicians and lead to delay in diagnosis. Therefore, it is associated with serious maternal morbidity. We present two cases of acute postpartum HELLP syndrome after caesarean section following severe preeclampsia. Our cases were successfully managed with the timely diagnosis and therapy.

## 1. Introduction

HELLP is an acronym that refers to a syndrome characterized by hemolysis, elevated liver enzymes, and a low platelet count [1]. It occurs in 0.5%–0.9% of all pregnancies. About 70% of the cases happen before delivery and the remainder within 48 hours after delivery. Although there are no differences in laboratory findings between HELLP syndrome before and after delivery, women with postpartum HELLP syndrome have significantly higher incidences of complications, such as pulmonary edema, renal failure, disseminated intravascular coagulation, and subcapsular liver haematoma [2, 3]. Because of absence of classical signs of preeclampsia, it can confuse physicians and lead to delay in diagnosis. HELLP syndrome is associated with serious maternal morbidity, especially when it arises in the postpartum period. We present two cases of acute postpartum HELLP syndrome after caesarean section following severe preeclampsia.

## 2. Case 1

A 43-year-old woman who was 30 weeks pregnant (Gravid 4, Para 3) presented in our hospital with a ten-day history of low extremities edema, with idiopathic hypertension for 1 year. She had never experienced discomfortable symptoms before, so she was taking no antihypertensive drugs. There were no complaints of nausea or vomiting and upper quadrant abdominal pain. At presentation, she had a blood pressure of 150/100 mmHg. Laboratory findings were normal except urinalysis (protein 2+). She was diagnosed with the chronic hypertension supervose severe preeclampsia. It was decided to deliver the fetus by means of a caesarian section. A Pfannenstiel incision was made and a fetus was delivered. After the operation, intravenous magnesium sulfate and antihypertension drug were given to the patient. Blood pressure measurement was 150/100 mmHg. She lost consciousness for 30 seconds five hours after operation. The laboratory studies gave the following results: serum aspartate aminotransaminase (AST), 225 IU/L; serum alanine aminotransaminase (ALT), 140 IU/L; serum lactate dehydrogenase (LDH), 1017 IU/L; serum urea and creatine were normal; hemoglobin, 10.6 mg/dL; platelet count, 50 × 10^3^ *μ*/mL. A brain computed tomography (CT) scan was performed on patient which revealed the left frontal lobe lacunar infarction. The patient was transferred to intensive care unit. After transfer to intensive care unit, two units of packed red blood cells and 6 units of fresh-frozen plasma were transfused to her. Antihypertensive treatment and corticosteroids had been given to the patient. Eight days postpartum, the patient had no complaint. She was referred to the cardiovascular service for better antihypertensive control and was subsequently discharged from the hospital in a stable condition (Table 1).

## 3. Case 2

A 22-year-old woman was admitted to our department because of loss of consciousness after caesarean section. This patient term pregnant (Gravid 1, Para 1) presented in other hospital. She had never experienced discomfortable symptoms in this pregnancy. At presentation, she had a blood pressure of 150/100 mmHg. Laboratory findings were normal except urinalysis (protein 3+). She had emergency operation because of severe preeclampsia and fetal distress. After the operation, intravenous magnesium sulfate and antihypertension drug were given to the patient. Blood pressure measurement was 150/100 mmHg. There were complaints of nausea or vomiting and upper quadrant abdominal pain 3 hours after operation. She had convulsion and loss of consciousness for four hours after operation. The laboratory studies gave the following results: serum aspartate aminotransaminase (AST), 263 IU/L; serum alanine aminotransaminase (ALT), 203 IU/L. The patient was transferred to our hospital. The laboratory studies gave the following results: serum aspartate aminotransaminase (AST), 306 IU/L; serum alanine aminotransaminase (ALT), 224 IU/L; serum lactate dehydrogenase (LDH), 2260 IU/L; serum urea and creatine were normal; hemoglobin, 18.6 mg/dL; platelet count, 34 × 10^3^ *μ*/mL. A brain MRI was performed on patient which revealed the eclampsia encephalopathy; two units of packed red blood cells, antihypertensive treatment, and corticosteroids had been given to the patient. Nine days postpartum, the patient had no complaint. She was discharged from the hospital in a stable condition [4] (Table 2).

## 4. Discussion

HELLP is a multisystem disease, resulting in generalized vasospasm, microthrombi formation, and coagulation defects. The syndrome seems to be the final manifestation of insult that leads to microvascular endothelial damage and intravascular platelet aggregation. Up to 30% of all patients who develop HELLP syndrome will develop this syndrome after parturition, typically within 48 hours. Unexpectedness, suddenness, and fulminant course of this syndrome are essential. The usual short period of observation after an uncomplicated delivery and uneventful medical history contribute to the risk of missing a life-threatening complication.

The most important symptom of this syndrome is epigastric pain which is assumed to be caused by stretching of Glisson's capsule due to sinusoidal obstruction of blood flow. However, such nonspecific abdominal symptom may confuse physicians and lead to diagnostic delay. Clinically evident DIC as a secondary pathophysiological phenomenon to the primary process is seen in 4%–38% of the patients, suggesting an important role of coagulopathy in the aetiology of HELLP syndrome. The development of a decompensation of coagulation correlates with the appearance of severe maternal complications such as renal failure.

It is unusual but a life-threatening complication. The HELLP syndrome usually occurs with preeclampsia, like in our patients which was not regularly controlled. All laboratory findings were normal. With regard to the forensic aspects of a timely diagnosis, an absence of obvious signs of preeclampsia significantly affected the clinicians who could not predict the development of HELLP syndrome, so timely and early diagnosis of HELLP is important.

In summary, we present two cases of postpartum HELLP syndrome. Our cases were successfully managed with the timely diagnosis and therapy.

## Figures and Tables

**Table 1:** Laboratory findings postpartum in case 1. Time postpartum.

Laboratory findings	1 d	2 d	3 d	4 d	5 d	6 d	7 d
Hemoglobin (g/L)	137	106	57	69	93	95	108
Platelet count (10^9^/L)	181	50	74	98	143	75	224
Total bilirubin (mmol/L)	7.3	19.2	10.4	8.2	7.6	7.5	9.2
Direct bilirubin (mmol/L)	1.3	6.1	2.7	2.2	1.9	1.6	1.7
Indirect bilirubin (mmol/L)	6.0	13.1	7.7	6.0	5.7	5.9	7.5
AST (IU/L)	14	225	83	35	41	21	15
ALT (IU/L)	16	140	97	53	58	46	36
LDH (IU/L)	293	1017	397				

**Table 2:** Laboratory findings postpartum in case 2. Time postpartum.

Laboratory findings	1 d	2 d	4 d	5 d	7 d
Hemoglobin (g/L)	186	153	127	124	124
Platelet count (10^9^/L)	34	28	115	138	214
Total bilirubin (mmol/L)	43.9	19.7	10.0	9.0	7.0
Direct bilirubin (mmol/L)	15.6	17.3	2.0	3.8	2.4
Indirect bilirubin (mmol/L)	28.3	12.4	8.0	5.2	4.6
AST (IU/L)	306	80.6	48	48	31
ALT (IU/L)	224	131	92	104	90
LDH (IU/L)	2260	1051	535	418	
